# Downregulation of A disintegrin and metallopeptidase with thrombospondin motif type 1 by DNA hypermethylation in human gastric cancer

**DOI:** 10.3892/mmr.2015.3667

**Published:** 2015-04-23

**Authors:** JING CHEN, CHUNDONG ZHANG, XIAOYANG XU, XINJIANG ZHU, DONGQIU DAI

**Affiliations:** Department of Gastrointestinal Surgery, The Fourth Affiliated Hospital of China Medical University, Shenyang, Liaoning 110032, P.R. China

**Keywords:** A disintegrin and metallopeptidase with thrombospondin motif type 1, angiogenesis, methylation, gastric cancer

## Abstract

A disintegrin and metallopeptidase with thrombospondin motif type 1 (ADAMTS1) is a metalloproteinase with antiangiogenic activity. It was previously observed that the mRNA and protein levels of ADAMTS1 are downregulated in primary gastric tumors. The aim of the present study was to examine whether the reduction in the expression of ADAMTS1 is due to aberrant methylation of the gene in primary gastric tumor tissues and gastric cancer cell lines. In addition, the association between ADAMTS1 methylation and clinicopathological features in were investigated in patients with primary gastric cancer. The results revealed that the frequency of ADAMTS1 methylation in primary gastric tumor tissues was significantly higher, compared with the corresponding normal gastric tissues. The relative mRNA expression levels of ADAMTS1 were significantly lower in the methylated primary gastric tumor tissues, compared with the unmethylated primary gastric tumor tissuess. A significant association was observed between the ADAMTS1 methylation status and the depth of tumor invasion and tumor, node, metastasis stage in primary gastric cancer. The mRNA expression of ADAMTS1 was significantly lower in 60% (3 of 5) of the gastric cancer cell lines. The relative mRNA expression levels of ADAMTS1 were significantly lower in the methylated gastric cancer cell lines, compared with the unmethylated gastric cancer cell lines. Furthermore, the expression of ADAMTS1 was significantly restored following treatment with the 5-Aza-2′-deoxycytidine demethylating agent in the MGC-803, HGC-27 and AGS gastric cancer cell lines, and the demethylation of the MGC-803 cell line inhibited cell invasion. Together, these results suggested for the first time, to the best of our knowledge, ADAMTS1 as a novel antitumor protease, and this function was lost following epigenetic silencing in the gastric cancer cells and gastric tumor tissues. Therefore, the aberrant methylation of ADAMTS1 may be involved in the development and progression of gastric cancer.

## Introduction

A disintegrin and metallopeptidase with thrombospondin motifs (ADAMTS) is a matrix metalloproteinase, which consists of multiple domains, including propeptide, metalloproteinase, disintegrin, spacer region domains, and domains containing thrombospondin type I (TSP-I) motifs (1). The ADAMTS family contains 19 members. ADAMTS are characterized by the presence of additional TSP-I motifs at their C-terminal region, while epidermal growth factor-like, transmembrane and cytoplasmic domains are missing (2,3). Several of these genes are involved in a variety of human diseases, including connective tissue disorder, inflammation, arthritis, cancer and thrombotic thrombocytopenic purport (4–10). Certain ADAMTS are involved in the progression of cancer, including cell proliferation, apoptosis, migration, invasion and angiogenesis (11). Although the precise role of ADAMTS in the development and progression of cancer remains to be elucidated, previous studies have provided evidence of dysregulation of certain ADAMTS in different types of cancer.

ADAMTS1, the first described member of the ADAMTS family, is widely expressed in various human tissues (1,12). Previous studies have demonstrated that ADAMTS1 can degrade aggrecan and versican, which are components of extracellular matrix (ECM) barriers (13,14). It is also considered to be a potent anti-angiogenic factor, which inhibits endothelial cell proliferation and angiogenesis (15,16). ADAMTS1 inhibits angiogenesis through binding to vascular endothelial growth factor (VEGF) 165 and inhibiting VEGF-A165-stimulated phosphorylation of VEGFR-2 (17). In addition, it has decreased expression levels in a number of types of cancer (8,18–20), however, the underlying mechanism remains to be elucidated.

Gastric cancer is considered to be caused by multiple factorS and develop in a multistep process. Epigenetics, particularly DNA methylation, has been investigated as one of the causes of the progression and development of gastric cancer (21). Several studies have indicated that DNA hypermethylation is an important mechanism in the transcriptional silencing of tumor-associated genes in gastric cancer. Our previous study demonstrated that decreased expression of ADAMTS1 is observed in primary gastric cancer (22). This suggests the possibility that the loss of ADAMTS1 function may be an important factor in tumor angiogenesis. To further examine the relevance of ADAMTS1 as a putative tumor-suppressor enzyme, the present study aimed to analyze the epigenetic mechanisms controlling its expression in gastric cancer cells and tissues. To the best of our knowledge, no previous studies have investigated whether the abnormal gene expression of ADAMTS1 in primary gastric cancer is due to epigenetic alterations in the pattern of DNA methylation. The present study aimed to examine the expression of ADAMTS1 in gastric cancer and its association with aberrant methylation.

## Materials and methods

### Tissues samples

Paired normal and tumor samples were obtained from 56 patients with gastric cancer, who underwent curative resection between March 2009 and October 2009, at The Fourth Affiliated Hospital of China Medical University. None of these patients had undergone chemotherapy or radiotherapy prior to surgery. The patient group consisted of 41 males (73.2%) and 15 females (26.8%) with a mean age of 59.8 years (range, 30–89 years). All tissues were frozen in liquid nitrogen immediately following surgery and stored at −80°C until genomic DNA preparation. The present study was approved by the ethics committee of the Fourth Affiliated Hospital of China Medical University (Shenyang, China), and written informed consent was obtained from all patients.

### Cell lines

The SGC-7901, BGC-823, MGC-803, HGC-27 and AGS human gastric cancer cell lines and GES-1 immortalized normal gastric cell line were cultured in RPMI-1640 (Gibco Life Technologies, Carlsbad, CA, USA), supplemented with 10% fetal bovine serum (Gibco Life Technologies), penicillin (100 IU/ml) and streptomycin (100 *µ*g/ml) (Sangon Biotech Co., Ltd., Shanghai, China), and incubated in a humidified incubator containing 5% CO_2_ at 37°C. The SGC-7901, BGC-823, MGC-803, HGC-27 and AGS cells were obtained from the Institute of Biochemistry and Cell Biology, Chinese Academy of Sciences (Shanghai, China). The GES-1 cell was obtained from the Oncology Institute of China Medical University (Shenyang, China).

### RNA isolation and reverse-transcription-quantitative poly-merase chain reaction (RT-qPCR)

The total RNA from the cultured cells (density, 5×10^6^) were isolated using TRIzol reagent (Invitrogen Life Technologies, Carlsbad, CA, USA). The DNase I-treated (1 *µ*l; 37°C for 45 min; Fermentas, Vilnius, Lithuania) total RNA (2 *µ*g) was converted to cDNA using the RevertAid First Strand cDNA Synthesis kit (Fermentas). The reverse transcriptase reaction was, as described previously (23). The primers for the human *ADAMTS1* gene were forward 5′-AAGCTGCTGATGGCACATATATTCA-3′ and reverse 5′-TTTTAGGTCGAAGGGCATTGC-3′, which generated a 195 base pair (bp) fragment. GAPDH was also amplified as an internal control using the following primers: forward 5′-GAAGGTCGGAGTCAACGGAT-3′ and reverse 5′-CCTGGAAGATGGTGATGGGAT-3′, which generated a 224 bp fragment. All primers were obtained from Sangon Biotech Co., Ltd. The qPCR was performed with the PTC-100 cyling machine (MJ Research, Inc., Waltham, MA, USA) in a volume of 50 *µ*l, containing 10X DreamTaq buffer (Fermentas), 2 mM dNTP Mix, 0.4 *µ*M each primer and 1.25 units DreamTaq. Initial denaturation was set at 95°C for 5 min, followed by 35 cycles of denaturation at 95°C for 20 sec, an annealing step at 58°C for 20 sec and an extension step at 72°C for 30 sec. A final extension step at 72°C for 5 min was performed. The qPCR products were separated on 1.5% agarose gels and quantified using a Fluor Chem 2.0 system (Bio-Rad Laboratories, Inc., Hercules, CA, USA). The mRNA levels were determined by quantifying the intensities of the qPCR products against GAPDH. Low and high mRNA levels were classified by the estimated value below and above the median value.

### Genomic DNA isolation and methylation-specific PCR (MSP)

The genomic DNA was prepared from the cell lines and fresh tissues using a phenol/chloroform procedure and was modified using bisulfite, as described previously (24). The DNA (2 *µ*g) was purified using a Wizard DNA Clean-Up system (Promega Corporation, Madison, USA), precipitated with 100% ethanol (Sangon Biotech Co., Ltd.) and resuspended in 30 *µ*l Tris-EDTA buffer (Sigma-Aldrich, St. Louis, MO, USA). The aliquot (2 *µ*l) was used as a template and 2 *µ*g DNA from each sample was treated with sodium bisulfite (Sigma-Aldrich) at 50°C for 16 h. The DNA was then washed with 70% ethanol and dissolved in 15 *µ*l TE. The methylated primer sequences were: Forward 5′-CGTTAGGTATTAATTTTCGC-3′ and reverse 5′-CGCTACAATTCTACCGACGCG-3′ (151 bp); the unmethylated primer was: Forward 5′-TGT TAG GTAT TA AT T T T TGT-3′ a nd reverse 5′-CACTACAATTCTACCAACACA-3′ (151 bp) (25). The reaction mixture contained 2 *µ*l DNA in a volume of 50 *µ*l, containing 2 *µ*l of each primer, 10X DreamTaq buffer, 2.0 mM dNTP mix and 1.25 units DreamTaq (Fermentas.) The complete MSP conditions were as follows: 94°C for 5 min, 94°C for 30 sec, 40 cycles of 72°C for 45 sec, 56°C for 30 sec, 72°C for 30 sec and 72°C for 10 min. Human placental DNA, treated *in vitro* with Sss I methylase via MSP (New England Biolabs, Inc. Ipswich, MA, USA), served as a positive control for the methylated reaction. Water was used as a negative PCR control. The MSP products were separated using electrophoresis through a 2.5% agarose gel containing ethidium bromide, and were quantified using a Fluor Chem 2.0 system. Each MSP was repeated at least once to confirm the results.

### Treatment of cells with 5-Aza-2′-deoxycytidine (5-Aza-dC)

The six cell lines (density, 5×10^6^) were incubated in culture media (RPMI-1640) at room temperature with 5 *µ*M 5-Aza-dC demethylating agent (Sigma-Aldrich) for 3 days. Furthermore, the MGC-803 cells were incubated in culture media with 2.5, 5 or 10 *µ*M of 5-Aza-dC for 3 days, with daily media replacement. These cells were then analyzed using RT-PCR, with the MGC-803 cells also analyzed using immunofuorescence on the third day.

### Immunofluorescence

Indirect immunofluorescence was performed on the ADAMTS1 cells grown in 24-well plates (Costar Corporation, Corning, NY, USA). The MGC-803 cells were fixed in 4% paraformaldehyde (Sangon Biotech Co., Ltd.) at 25°C for 15 min, permeabilized with 0.2% Triton X-100 (Sigma-Aldrich) and then washed three times with phosphate-buffered saline (PBS). The cells were incubated with rabbit anti-human ADAMTS1 polyclonal antibody (1:400; ab39194; Abcam, Cambridge, UK) overnight at 4°C, followed by incubation in the dark at room temperature with a 1:100 dilution of goat anti-rabbit rhodamine-conjugated immunoblobulin G-R (sc-2091; Santa Cruz Biotechnology, Inc., Santa Cruz, CA, USA) for 2 h. The nuclei were visualized using Hoechst 33258 counterstaining (Sangon Biotech Co., Ltd.) and examined using a fluorescence microscope (Olympus BX-40; Olympus, Tokyp, Japan).

### Matrigel invasion assay

An invasion assay was performed using an 8 *µ*m pore size membrane coated with Matrigel (Corning Life Sciences). The MGC-803 cells (2×10^4^) in 200 *µ*l serum-free 1640 media were seeded onto Matrigel-coated Transwell filters (Corning Life Sciences) in the upper chambers. The lower chambers contained 500 *µ*l RPMI-1640 medium (Invitrogen Life Technologies) with 10% FBS to serve as a chemoattractant, and the chambers were incubated at 37°C in a humidified 5% CO_2_. After 24 h, the non-invading cells on the upper membrane surface were removed using cotton swabs. The cells on the lower side of the membrane (invading cells) were fixed in 4% paraformaldehyde for 30 min and then stained with methylrosanilinium chloride (Sangon Biotech Co., Ltd.). The invaded cells were viewed under the Olympus BX-40 microscope and counted in five randomly selected fields (magnification, x200). The invasion abilities of the cancer cells were expressed as the mean number of cells in the five fields. The assay was performed in three independent experiments.

### Data analysis

Statistical analysis was performed using the SPSS statistical software package, version 17.0 (SPSS, Inc., Chicago, IL, USA). The χ^2^ test was used to determine the association between ADAMTS1 methylation and the clinicopathological features (Table I). In addition, the non-parametric Mann-Whitney U test was used to determine the relative mRNA levels of ADAMTS1 in the cell lines, and their association with methylation. P<0.05 was considered to indicate a statistically significant difference.

## Results

### mRNA expression of ADAMTS1 in cell lines

**Figure 1:**
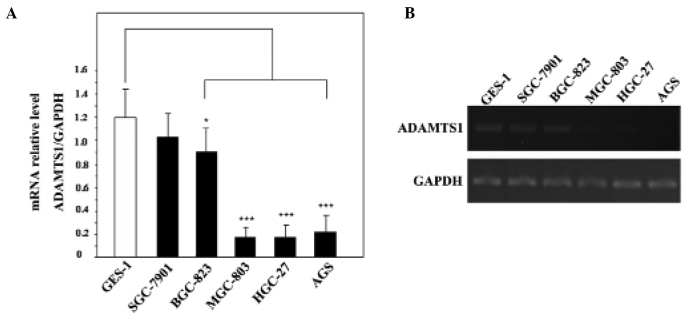
mRNA expression levels of ADAMTS1 in six gastric cell lines. (A) mRNA eof ADAMTS1 in the GES-1 normal gastric cell line and five gastric cancer cell lines. GAPDH was used as a control. (B) Relative mRNA expression levels of ADAMTS1 in the GES-1 normal gastric cell line and five gastric cancer cell lines. (Mann-Whitney U test; ^*^P<0.05 and ^***^P<0.001, compared with the GES-1 cell line). Data are presented as the mean ± standard deviation. ADAMTS1, A disintegrin and metallopeptidase with thrombospondin motif type 1.

Representative examples are shown in Fig. 1A. The expression of ADAMTS1 was undetectable in the MGC-803, HGC-27 and AGS gastric cancer cell lines of the six cell lines examined. Statistically, the mRNA expression of ADAMTS1 was significantly lower in the BGC-823, MGC-803, HGC-27 and AGS gastric cancer cell lines, compared with the GES-1 normal gastric cell line. (P=0.022, P=0.000, P=0.000 and P=0.000, respectively; Fig. 1B).

### Aberrant methylation of ADAMTS1 in the cell lines

**Figure 2:**
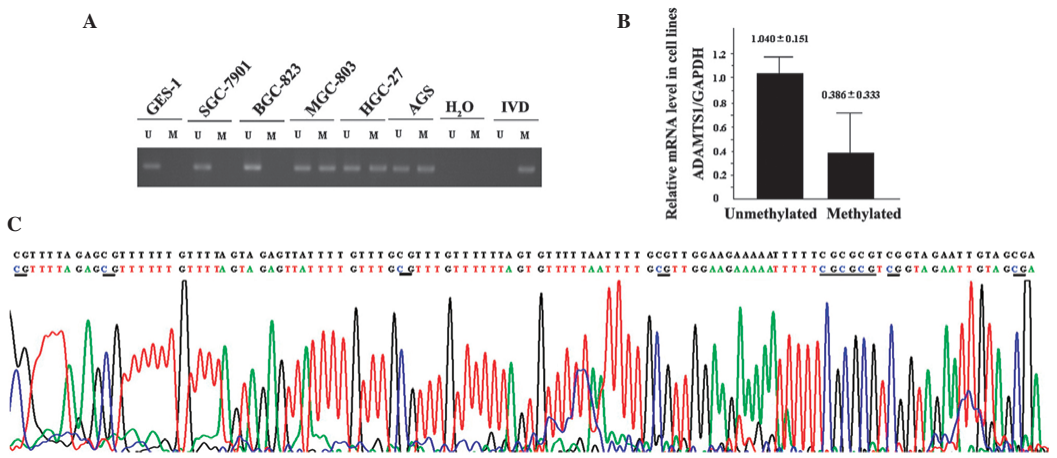
Methylation analysis of ADAMTS1 in the six gastric cell lines. (A) Methylation status of ADAMTS1 in the GES-1 normal gastric cell line and five gastric cancer cell lines. (B) Relative mRNA levels of ADAMTS1 in the unmethylated gastric cell lines and methylated gastric cell lines (Mann-Whitney U test; P=0.036). (C) Bisulphite sequence electropherogram of methylated MGC-803. CG, which remained unchanged are underlined in black. Data are presented as the mean ± standard deviation. U, unmethylated; M, methylated; IVD, positive control; H_2_O, negative control; ADAMTS1, A disintegrin and metallopeptidase with thrombospondin motif type 1.

The status of DNA methylation in the six cell lines was examined. Methylation was observed in the MGC-803, HGC-27 and AGS, gastric cancer cell lines, whereas no methylation was observed in the SGC-7901 and BGC-823 gastric cancer cell lines or the GES-1 normal gastric cell line (Fig. 2A). The statistical analyses demonstrated that the relative mRNA levels of ADAMTS1 were significantly lower in the methylated gastric cancer cell lines, compared with the unmethylated gastric cancer cell lines (P=0.036; Fig. 2B) Furthermore, the promoter methylation sequence of ADAMTS1 was detected following treatment with sodium bisulfate in the methylated MGC-803 cell line, and all the CG bases remained as CG bases, which confirmed that the ADAMTS1 promoter was hypermethylated in the MGC-803 cell line (Fig. 2C).

### mRNA and protein expression levels of ADAMTS1 in cell lines following treatment with 5-Aza-dC

**Figure 3:**
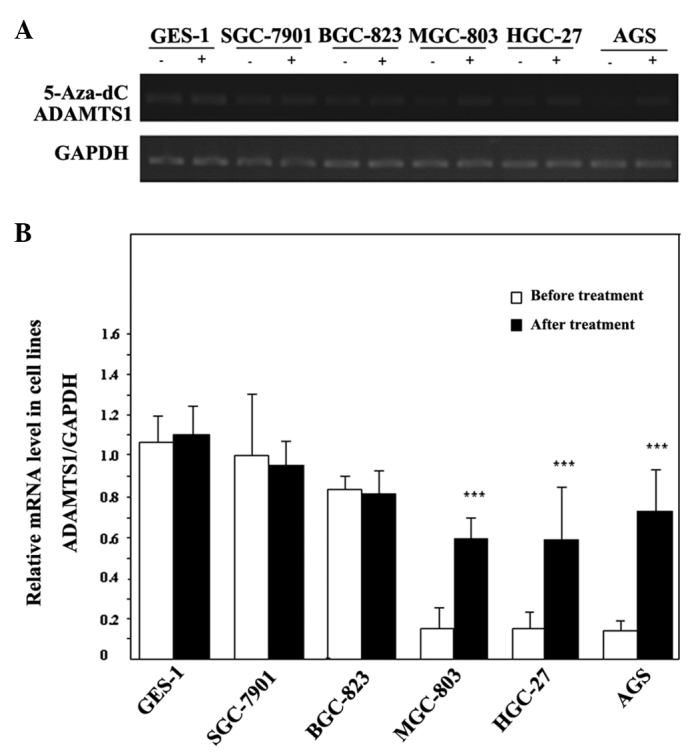
mRNA expression levels of ADAMTS1 in gastric cell lines before and after treatment with 5-Aza-dC. (A) mRNA expression levels of ADAMTS1 in the GES-1 normal gastric cell line and five gastric cancer cell lines were analyzed using reverse transcription-quantitative polymerase chain reaction analysis. (B) Relative mRNA expression levels of ADAMTS1 in the GES-1 normal gastric cell line and five gastric cancer cell lines (Mann-Whitney U test; ^***^P<0.001, vs. before treatment). Data are presented as the mean ± standard deviation. ADAMTS1, A disintegrin and metallopeptidase with thrombospondin motif type 1; 5-Aza-dC, 5-Aza-2′-deoxycytidine.

**Figure 4:**
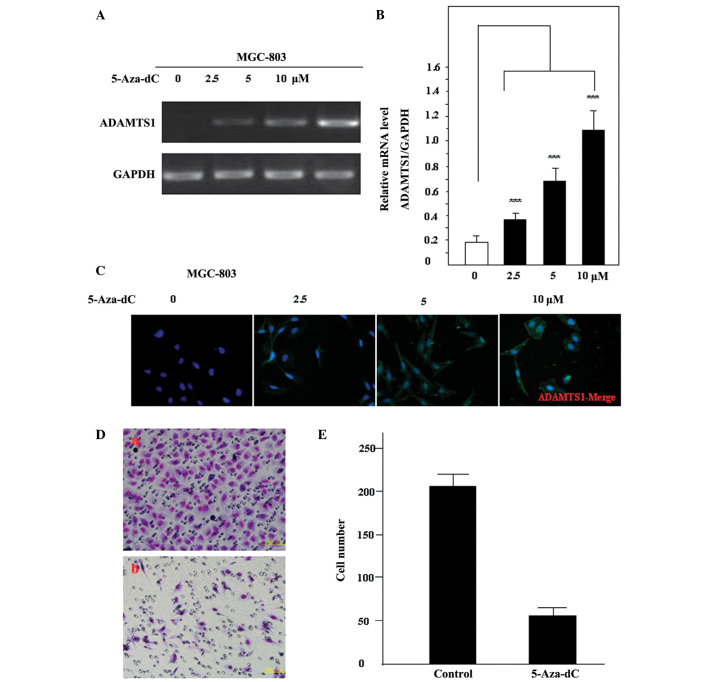
mRNA and protein expression levels of ADAMTS1 in the MGC-803 cell line prior to and following treatment with different concentrations of 5-Aza-dC for 3 days. (A) mRNA expression of ADAMTS1 prior to and following treatment with different concentrations of 5-Aza-dC using reverse transcription-quantitative polymerase chain reaction analysis. (B) ADAMTS1 relative mRNA levels prior to and following treatment with different concentrations of 5-Aza-dC (Mann-Whitney U test; ^***^P<0.001). (C) Protein levels of ADAMTS1 prior to and following treatment with different concentrations of 5-Aza-dC, detected using immunofuorescence (magnification, x200). The MGC-803 cells immunostained with antibody to ADAMTS1 (green) were characterized by perinuclear cytoplasmic immunofluorescence. The cell nuclei were visualized with Hoechst33258 (blue). (D) Representative figures of MGC-803 cell invasion in untreated and 5-Aza-dC-treated MGC-803 cells. (a) Invasion in the untreated group; (b) invasion in the 5-Aza-dC-treated group. (E) Numbers of MGC-803 cells following treatment with 5-Aza-dC compared with the untreated control group. The values are presented as the mean ± standard deviation (Mann-Whitney U test; P<0.001). ADAMTS1, A disintegrin and metallopeptidase with thrombospondin motif type 1; 5-Aza-dC, 5-Aza-2′-deoxycytidine.

In the present study, the six cell lines were treated with 5-Aza-dC, which resulted in restoration of the mRNA expression of ADAMTS1 in the MGC-803, HGC-27 and AGS cell lines, determined using RT-qPCR analysis (Fig. 3A). As shown in Fig. 3B, the relative mRNA levels of ADAMTS1 were significantly higher following treatment with 5-Aza-dC in the MGC-803, HGC-27 and AGS cell lines, compared with those prior to treatment with 5-Aza-dC (P<0.001). The MGC-803 cell line was then treated for 3 days with 2.5, 5 or 10 *µ*M 5-Aza-dC. The results revealed that the mRNA expression of ADAMTS1 was significantly upregulated following 5-Aza-dC treatment (Fig. 4A). In addition, the effect of 5-Aza-dC on the expression of ADAMTS1 was dose-dependent, with higher doses resulting in increased changes in the expression of ADAMTS1 (Fig. 4B). The present study also examined the protein expression of ADAMTS1 using immunofluorescence, and observed a marked upregulation in ADAMTS1 (Fig. 4C).

### Inhibition of MGC-803 cell invasion following treatment with 5-Aza-dC

The invasive properties of the MGC-803 were affected by treatment with 10 *µ*M 5-Aza-dC for 3 days. The results demonstrated that the number of invading MGC-803 cells was significantly reduced following 5-Aza-dC treatment, compared with the control group (51±8.47 vs. 206±18.11; P<0.001; Fig. 4D and E).

### ADAMTS1 methylation in gastric cancer and clinicopathologic correlations

**Figure 5:**
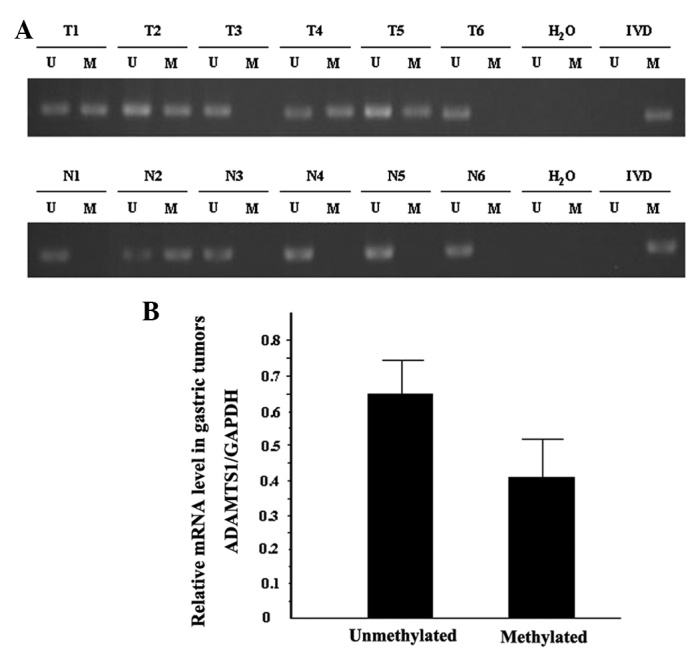
Methylation analysis of ADAMTS1 in gastric tissue samples. (A) Methylation of ADAMTS1 in primary gastric tumor tissues and corresponding normal gastric tissues. (B) Relative mRNA levels of ADAMTS1 in the unmethylated primary gastric tumor tissues and methylated primary gastric tumor tissues (Mann-Whitney U test; P=0.043). Data are presented as the mean ± standard deviation. N, normal tissue; T, tumor tissue; U, unmethylated; M, methylated; IVD, positive control; H_2_O, negative control. ADAMTS1, A disintegrin and metallopeptidase with thrombospondin motif type 1.

Our previous study reported low mRNA expression levels of ADAMTS1 in 56 primary gastric tumors. In the present study, the methylation status of the ADAMTS1 promoter was assessed in these primary gastric tumor tissues and in normal gastric tissues using MSP. Representative examples of the MSP analysis are shown in Fig. 5A. Methylation of the ADAMTS1 gene was detected in 27 (48%) primary gastric tumor tissues and eight (14%) corresponding normal gastric tissues. Methylation of the ADAMTS1 gene was significant higher in primary gastric tumor tissues compared with normal gastric tissues (P=0.009). In addition the relative mRNA levels of ADAMTS1 were significantly lower in the methylated primary gastric tumor tissues compared with those in the unmethylated primary gastric tumor tissues (P=0.043; Fig. 5B). The present study also investigated the association between ADAMTS1 methylation and the clinicopathological features of the patients, including age, gender, size, differentiation, depth of tumor invasion, tumor, node, metastasis (TNM) Stage and lymph node metastasis. A significant association was observed between the ADAMTS1 methylation status, depth of tumor invasion and TNM stage. Methylation was significantly more frequent in primary tumor tissues at the pT3-pT4 stages, compared with those with at the pT1-pT2 stages (P=0.048). Methylation was significantly more frequent in primary tumor tissues at the III–IV stage, compared with those with at the I–II stages (P=0.015). None of the other clinicopathological factors were significantly associated with ADAMTS1 methylation (Table I).

## Discussion

The present study investigated the mRNA expression of ADAMTS1 in five human gastric cancer cell lines and one immortalized normal gastric cell line, and analyzed the methylation status of ADAMTS1 using MSP in the cell lines and primary gastric tumor tissues. Furthermore, the present study also examined whether the loss of the expression of ADAMTS1 ws due to aberrant methylation of the gene. To the best of our knowledge, the present study is the first concerning the expression methylation status of ADAMTS1 in human gastric cancer.

Although the functional roles of ADAMTS1 in carcinogenesis remain to be fully elucidated, the anti-angiogenic properties of ADAMTS1 have been described. A previous report suggested that ADAMTS1 gene transfer inhibited angiogenesis *in vitro* and *in vivo*, possibly as a result of the induction of endothelial cell apoptosis by ADAMTS1, which occurs independently of protease activity (16). It sequesters the potent angiogenic stimulant, VEGF, from interacting with its receptors, preventing an angiogenic effect. ADAMTS1 also cleaves large TSP1, and cleaved TSP-1 demonstrates anti-angiogenic activity (26). Several studies have also demonstrated that ADAMTS1 is downregulated in breast cancer, prostate cancer, lung cancer, pancreatic cancer and colorectal cancer (18–20,25,27,28). These findings are in accordance with our previous study, in which the expression of ADAMTS1 was frequently reduced in primary gastric cancer (22). In the present study, the mRNA expression of ADAMTS1 was significantly lower in the gastric cancer cell lines compared with the normal gastric cell line. Together, these findings support the hypothesis that ADAMTS1 functions as a tumor inhibitor in the gastric cancer. Inversely, ADAMTS1 protein can exert pro- and anti-angiogenic properties, dependent upon its proteolytic status (29). Several reports have revealed that the expression of ADAMTS1 was elevated in endometrial adenocarcinoma (10) and breast cancer (30). Metastatic cancer was often associated with increased ADAMTS1 (29,31). These findings suggest the binary role of ADAMTS1 in cancer.

It has been reported that the expression of ADAMTS1 is epigenetically silenced in non-small cell lung cancer (NSCLC) (25,32), prostate cancer (33) and colorectal tumor tissues (28,34), however, no significant association has been observed between hypermethylation and reduced expression of ADAMTS1 in colorectal tumor tissues (34). Methylation in NSCLC tumor tissues is not correlated with clinicopathologic features of the patients, including age, gender, histology and pathologic staging (25). In addition, several studies have revealed that the ADAMTS9 and ADAMTS18 gene promoters are also hypermethylated in gastric carcinoma (35,36). There are no previous studies describing the methylation status of ADAMTS1 in human gastric tumor tissues. The present study demonstrated that the frequency of ADAMTS1 methylation in primary gastric tumors and gastric cancer cell lines was significantly higher than in normal gastric tissues and the normal gastric cell line. In addition, the downregulation in the expression of ADAMTS1 was correlated with methylation of its promoter in the gastric cancer cell lines and gastric cancer tissues. The results also revealed that tumors with an invasion depth at T3 and T4 had markedly higher methylation frequencies than those with an invasion depth at T1 and T2. The methylation frequency of ADAMTS1 was also negatively associated with TNM stage. These results suggested that aberrant methylation of ADAMTS1 was involved in the pathogenesis of gastric cancer, and the invasion ability of gastric cancer may be higher when the methylation frequency of ADAMTS1 is high.

5-Aza-dC, a demethylating agent, is a nucleoside analog, which can inhibit DNA cytosine methylation, including reactivation of epigenetically silenced genes (37). In the present study 5-Aza-dC reactivated the expression of ADAMTS1. This suggested that reactivation of silenced genes correlated with decreased DNA methylation. The effect of 5-Aza-dC treatment on cancer cell invasion was determined using a Matrigel invasion assay. The number of invading MGC-803 cells was significantly reduced following treatment with 5-Aza-dC, which indicated that 5-Aza-dC inhibited the invasive ability of the MGC-803 cells. These results revealed that DNA demethylation decreased the invasive force of the gastric cancer cells. An understanding of the precise mechanism of the down-=regulation of ADAMTS1 in gastric cancer needs requires further investigation.

In conclusion, the results of the present study demonstrated that the mRNA expression of ADAMTS1 was significantly lower in gastric cancer cell lines. Furthermore, the frequency of ADAMTS1 methylation was significantly higher in primary gastric tumor tissues than in gastric cancer cell lines. The downregulation in the expression of ADAMTS1 was correlated with its promoter methylation in the primary gastric tumors and gastric cancer cell lines. These results suggested that the anti-angiogenic function of ADAMTS1 is primarily important in primary gastric cancer. This function is lost by epigenetic silencing in tumor cells and tumor tissues. Aberrant methylation may be involved in the development and progression of gastric cancer.

## Figures and Tables

**Table I:** Methylation of A disintegrin and metallopeptidase with thrombospondin motif type 1 in primary gastric tumor tissues and its association with clinicopathological factors.

Clinicopathological factor	Number of patients	Methylated (%)	Unmethylated (%)	P-value
Gender				0.435
Male	41	19 (46.3)	22 (53.7)	
Female	15	8 (53.3)	7 (46.7)	
Age (years)				0.540
≥65	18	9 (50)	9 (50)	
<65	38	18 (47.4)	20 (52.6)	
Tumor size (cm)				0.124
≥3	34	19 (55.9)	15 (44.1)	
<3	22	8 (36.4)	14 (63.6)	
Differentiation				0.297
Good/moderate	28	12 (42.9)	16 (57.1)	
Poor	28	15 (53.6)	13 (46.4)	
Depth of tumor invasion				0.048
pT1–pT2	15	4 (26.7)	11 (73.3)	
pT3–pT4	41	23 (56.1)	18 (43.9)	
TNM stage				0.015
I–II	17	4 (23.5)	13 (76.5)	
III–IV	39	23 (59.0)	16 (41.0)	
Lymph node metastasis				0.165
Positive	42	18 (42.8)	24 (57.2)	
Negative	14	9 (64.3)	5 (35.7)	

TNM, tumor, node, metastasis.
